# The Influence of Genetics and Gene Polymorphism on Biological Complications for Dental Implant Survival: A Review

**DOI:** 10.1055/s-0044-1787820

**Published:** 2024-07-16

**Authors:** Marcella Santos Januzzi, Daniela Micheline dos Santos, Cleber Davi Del Rei Daltro Rosa, Karina Helga Leal Turcio, Clóvis Lamartine de Moraes Melo Neto, Maria Eduarda da Silva Fernandes, André Pinheiro de Magalhães Bertoz, Manuel Martin Adriazola Ique, Marcelo Coelho Goiato

**Affiliations:** 1 Department of Dental Materials and Prosthetics, School of Dentistry, São Paulo State University, Araçatuba, Brazil

**Keywords:** dental implants, genetics, genetic polymorphism, genetic risk factor, interleukins

## Abstract

The objective of this review is to expose the main genetic changes and genetic polymorphisms that may or may not be associated with greater susceptibility to reduced survival of dental implants and, consequently, to their loss. Case–control studies that fully portrayed the specific types of genetic polymorphisms that may be associated with dental implant failure were included by searching in the PubMed, Scopus, and Web of Science databases from 2010 to 2023. The following descriptors and their combinations in English were used to search for articles: “dental implants,” “bone genes,” “genetics,” “polymorphism genetics,” “genetic risk factor,” and “interleukin.” The initial search resulted in 107 results (PubMed *n* = 47, Scopus *n* = 14, and Web of Science *n* = 46). After a manual search, reviewing each article's title and abstract, and excluding duplicates, systematic reviews, and literature reviews, 30 articles were selected. After reading these 30 articles in full, 18 studies that did not describe the specific genetic polymorphism in relation to dental implant survival were excluded. Therefore, 12 articles were included in this review. The genetic polymorphisms of interleukin (IL)-1A, IL-1B, IL-4, IL-6, IL-10, IL-1 receptor antagonist, tumor necrosis factor-α, receptor activator of nuclear factor kappa B legend, and cluster of differentiation 14 were analyzed in the included studies. In seven of the studies, a statistically significant correlation between genetic polymorphisms and dental implant failure was observed. Of the polymorphisms studied, IL-1A (−899), IL-1B (+3954), and IL-4 (+33) showed a greater association with dental implant loss.

## Introduction

Implant-supported prostheses provide better stability, retention, esthetics, patient satisfaction, confidence, and self-esteem. 1 2 3 4 Dental implants have shown high long-term survival rates (from 85 to 100%). 1 3 4 5 6 7 8 Failures, however, may occur in the implant's osseointegration. 5 9 This process of osseointegration failure in dental implants can be broken down into two stages: early and late. Early osseointegration failure can be caused by things such as surgical trauma, contamination during the procedure, and bone quantity and quality. Late osseointegration failure can be caused by peri-implantitis and occlusal overload. 1 10 Furthermore, the genetic factor may be linked to the dental implant loss. 2

The term genetic polymorphism is used to describe a gene variant. Most polymorphisms are single nucleotide exchanges caused by mutations, which occur at a high frequency in the human genome. 4 5 8 When the promoter region of a gene is affected, it can result in a reduction or increase in gene expression, producing fewer or more proteins, respectively. 5 On the other hand, when the coding region of a gene is affected, an altered protein with a different function can be formed. 5 Thus, the polymorphism can affect the patient's immunological and inflammatory responses, generating a negative impact on osseointegration. 2 4 5

Altered immune system proteins generated by genetic polymorphisms can be released during the interaction between the host and pathogenic microorganisms. 5 11 This can lead to an exacerbated inflammatory response, causing bone destruction around the implant and osseointegration failure (peri-implantitis). 5 9 10 11

The objective of this review is to expose the main genetic changes and genetic polymorphisms that may or may not be associated with greater susceptibility to reduced survival of dental implants and, consequently, to their loss.

## Methods

The following descriptors and their combinations in English were used to search for articles from 2010 to 2023: “dental implants,” “bone genes,” “genetics,” “polymorphism genetics,” “genetic risk factor,” and “interleukin” in the PubMed, Scopus, and Web of Science databases, using the Boolean search strategy: ((dental implants) AND (bone genes OR genetics OR polymorphism genetics OR genetic risk factor)) AND (interleukin).

The inclusion criteria were: articles (case–control) in English, with human beings, that evaluated the specific types of genetic polymorphisms that could be associated with dental implant failure. The search was expanded as necessary, and references to included articles were included in this review when appropriate.

## Results

**Fig. 1:**
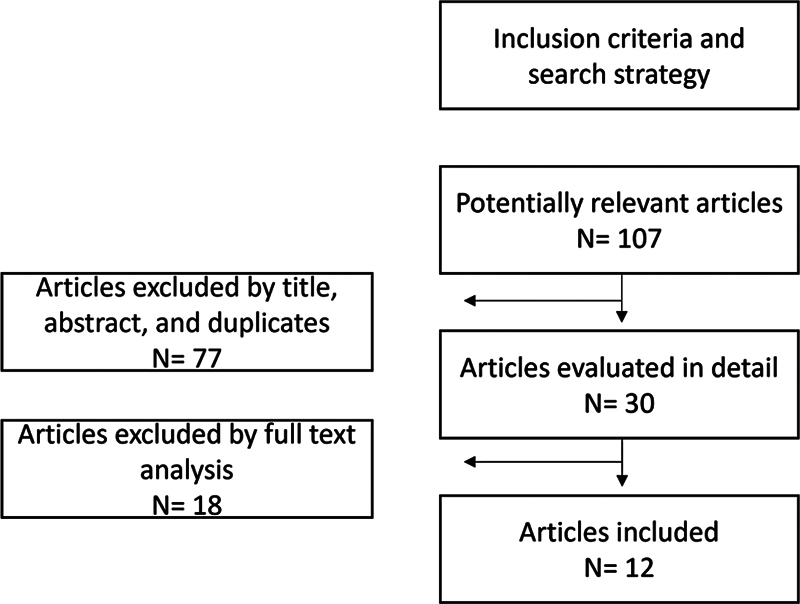
Search strategy and number of articles included in the research.

The initial search resulted in 107 results (PubMed *n* = 47, Scopus *n* = 14, Web of Science *n* = 46). After a manual search, reviewing each article's title and abstract, and excluding duplicates, systematic reviews, and literature reviews, 30 articles were selected. After reading these 30 articles in full, 18 studies that did not describe the specific genetic polymorphism in relation to dental implant survival were excluded. Therefore, 12 articles were included in this review. 1 2 3 4 5 6 7 8 9 10 11 12 The general screening process is detailed in Fig. 1 , and Table 1 represents the specifications of each of the articles.

**Table 1:** Specifications of the 12 case–control studies included in the review

Authors	Genetic polymorphisms studied	Considerations
Dirschnabel et al (2011) 4	IL-1B (−511)	Borderline association with dental implant loss
Gurol et al (2011) 12	IL-10 (−1082), IL-10 (−819), IL-10 (−592) and TNF-α (−308)	No association with dental implant loss
Hamdy and Ebrahem (2011) 11	IL-1A (−889), IL-1B (+3954)	Association with dental implant loss
Vaz et al (2012) 2	IL-1A (−889), IL-1B (+3953)	Association with dental implant loss
Melo et al (2012) 6	IL-1B (+3954), IL-1B (−511), and IL-6 (−174)	No association with dental implant loss
Pigossi et al (2012) 1	IL-10 (−1082), IL-10 (−819), and IL-10 (−592)	No association with dental implant loss
Pigossi et al (2014) 10	IL-4 (+33), IL-4 (−590)	Association with dental implant loss in IL-4 (+33)
Cosyn et al (2016) 5	IL-1A (−889), IL-1B (−511), and IL-1B (+3954)	Association with dental implant loss in IL-1A (−889) and IL-1B (+3954)
Petkovic-Curcin et al (2017) 7	TNF-α (−308), CD-14 (−159), IL-6 (−174), IL-10 (−1082), and IL-1ra	Association with dental implant loss (peri-implantitis) in TNF-α (−308), while CD-14 (−159) decrease the risk
Ribeiro et al (2017) 8	IL-10 (−1082), RANKL (−438)	No association with dental implant loss
Sampaio Fernandes et al (2017) 3	IL-1A (−889), IL-1B (+3953), and IL-1ra	Association with dental implant loss in IL-1B (+3953)
He et al (2020) 9	IL-1A (−889), IL-1B (+3954), and TNF-α (−308)	Association with dental implant loss in IL-1A (−889) and IL-1B (+3954)

Abbreviations: CD, cluster of differentiation; IL, interleukin; IL-1ra, IL-1 receptor antagonist; RANKL, receptor activator of nuclear factor kappa B legend; TNF, tumor necrosis factor.

### Characteristics of the Included Studies

Different categories of genes and their polymorphisms were analyzed, including interleukins (IL-1A [−889], IL-1B [+3954], IL-1B [+3953], IL-1B [−511], IL-4 [+33], IL-4 [−590], IL-6 [−174], IL-10 [−1082], IL-10 [−819], IL-10 [−592], IL-1 receptor antagonist [IL-1ra]), tumor necrosis factor (TNF-α [−308]), cluster of differentiation (CD-14 [−159]), and receptor activator of nuclear factor kappa B legend (RANKL [−438]).

The genetic polymorphisms of IL-1A (−889) were the subject of a study by five authors 2 3 5 9 11 who showed an association with biological complications for dental implant loss and, although present, were not statistically significant in only one of them. 3

The genetic alterations of IL-1B (−511) were reported in three articles 4 5 6 and the absence of an association with dental implant failure was observed in all of them, while IL-1B (+3954) was associated with the severity of progression of peri-implantitis and consequent reduction in implant survival in three of the four studies cited, 5 6 9 11 and was only negative in Melo et al. 6 IL-4 (+33) and IL-4 (−590) were only studied by Pigossi et al, 10 showing an association only in the first polymorphism reported. IL-6 (−174), 6 7 IL-10 (−1082), 1 7 8 12 IL-10 (−819), 1 12 and IL-10 (−592) 1 showed no statistically significant association with dental implant loss in any of the included studies. Of the remaining genes, CD-14 (−159), cited by Petkovic-Curcin et al, 7 showed a positive association with biological complications, while RANKL (−438), cited by Ribeiro et al, 8 did not have the same result, and TNF-α (−308) was associated with implant failure in one 7 of the three included studies. 7 9 12

## Review

The reduction in the survival of dental implants due to their failure is a consequence of a multifactorial process, and the clinical observation of repeated failures in the osseointegration of dental implants in specific patients raises doubts about the host's susceptibility to rehabilitation failure. 13 The patient's normal inflammatory immune response is critical for successful treatment, and genetic influences, such as genetic polymorphisms, can act as destructive or protective factors for a disease, altering the patient's immune response. 2 14 Genetic alterations causing greater IL activity would lead to greater alterations in bone metabolism, resulting in peri-implant bone loss and reduced rehabilitation survival, 15 involving different types of cells, such as macrophages, polymorphonuclear neutrophils, T and B lymphocytes, endothelial cells, fibroblasts, keratinocytes, osteoclasts, and osteoblasts. 1

Each type of IL plays an important role in the bone remodeling process, with IL-1 being a key mediator of inflammatory processes, which can promote the activation of the degradation cascade of extracellular matrix components through the induction of matrix metalloproteinases 4 and bone destruction through the interactions of RANK/osteoprotegerin, an important cytokine in activating the differentiation of monocytes into osteoclasts. 4 16

Of the 11 members of the IL-1 family, the proinflammatory proteins IL-1A and IL-1B seem to act as the most prominent protagonists of acute as well as chronic inflammation and have the ability to induce osteoblasts to secrete other ILs, 17 performing other functions, such as the anti-inflammatory activity triggered by IL-10 and the IL-1ra or proinflammatory activity such as IL-2, IL-6, and TNF-α, 2 4 7 as well as IL-4 acting as a potent down-regulator of macrophage function, inhibiting the secretion of proinflammatory cytokines. 18

Polymorphisms of the IL-1A (−889) and IL-1B (+3954) genes are the most studied in the literature, since these ILs and their respective positions are most commonly found in the hyperinflammatory response, with high production of these in the gingival sulcus in patients with a high-risk genotype. 19 20 Cosyn et al published a case–control study with the aim of exploring their impact on early implant failure. In a total of 14 healthy Caucasian patients with a history of failure of one or more dental implants within 6 months of installation, blood samples were taken, and genetic sequencing by polymerase chain reaction (PCR) was performed. The results report a significant impact of the IL-1A (−889) T allele ( *p* = 0.039) and the IL-1B (+3.954) T allele ( *p* = 0.003) on the onset of implant failure. In addition, the genotype distribution differed significantly between cases and controls for IL-1B (+3.954) ( *p* = 0.015), suggesting its relevance in osseointegration. 5

The results were similar to the study by Vaz et al, 2 who investigated a case–control study of 155 Portuguese Caucasian individuals, 100 of whom had successful rehabilitation and 55 of whom had dental implant failure, considering as failure the presence of mobility; pain on palpation, percussion, or function; recurrent infection; peri-implantitis; and exposure of the metal in the buccal area during or after abutment connection. The genetic test for periodontitis (TGP) was used as the methodology. The study confirmed that a positive TGP result was more associated with cases of failed implants, and a negative TGP result was more associated with successful cases. The role of these polymorphisms, especially IL-1B (+3953) ( *p* = 0.023), in the occurrence of biological complications such as pain, mobility, peri-implantitis, peri-implant mucositis, and dental implant loss was also mentioned by Sampaio Fernandes et al in an analysis of 229 implants from 58 patients with implant-retained overdentures. 3

Corroborating Cosyn et al, 5 Vaz et al, 2 Sampaio Fernandes et al, 3 and He et al 9 studied that individuals carrying the T allele of IL-1A (−889) ( *p* = 0.006) and IL-1B (+3954) ( *p* = 0.03) have an increased risk of peri-implantitis, based on logistic regression analysis in a nonsmoking Chinese population of 144 patients with peri-implantitis and 174 healthy control patients. 9 Other authors who also reported the association were Hamdy and Ebrahem, 11 who observed that polymorphisms in IL-1A (−889) and IL-1B (+3.954) can affect the results of treatment for peri-implantitis in genotype-positive individuals. To assess the genetic relationship with disease progression severity, 25 patients with peri-implantitis and 25 patients with healthy peri-implant tissue were analyzed using clinical and radiographic parameters. Epithelial cells from the oral mucosa of all patients were collected, and PCR was performed for IL genotyping. As a result, 17 patients in the peri-implantitis group were genotype positive (68%) and 5 patients in the healthy group were also genotype positive (20%), with a statistically significant difference observed between the two groups ( *p* < 0.05), with the first group having higher scores. Another relevant point of research to be added is the genetic influence on treatment response, since patients with peri-implantitis but with a negative genotype showed a better response to periodontal treatment compared with patients with a positive genotype. 11

The only study to show a negative result was by Melo et al, 6 who indicated no influence between this polymorphism (IL-1B [+3.954]) and rehabilitation failure. Forty-seven implants from 47 patients were examined (31 healthy implants, 16 implants with peri-implantitis, 31 healthy teeth from patients with healthy implants, and 16 healthy teeth from patients with peri-implantitis) by collecting fluid from the gingival sulcus and analyzing genomic DNA. It was concluded that there was no statistically significant difference in the concentration of IL-1B (+3.954) ( *p* = 0.0814) detected between the groups, and the genetic polymorphism studied had no influence on peri-implant disease. According to the authors, the absence of a significant difference could be a consequence of the very high standard deviation found or a reflection of a chronic marginal infection with slow progression. 21

Case–control studies involving other nucleotide positions in the genetic polymorphism of the IL-1 genotype have also been studied, in one of which IL-1B (−511T) was analyzed using PCR to check the genomic DNA of the oral mucosa in a sample of 277 patients, 92 of whom had multiple early dental implant losses, and the control group was made up of patients without implant loss for at least 6 months. 4 The association of IL-1B (−511T) was tested, and it was concluded that more studies are needed to analyze IL-1 haplotypes, given the borderline association ( *p* = 0.083) found in the relationship between genetic polymorphism and dental implant loss. This result differs from those obtained by Cosyn et al 5 and Melo et al, 6 who found no statistically significant association ( *p* = 0.392 and *p* > 0.05, respectively). In general, although there are numerous studies in the literature evaluating different types of cytokines, the concentration required to differentiate between a healthy site and the onset of pathological periodontal or peri-implant disease is unclear. 6 22

Petkovic-Curcin et al, in a Serbian population ( *n* = 98), investigated susceptibility to peri-implantitis through the polymorphisms of the CD-14 (−159), TNF-α −308), IL genes, IL-6 (−174) and IL-10 (−1082), and IL-1ra genes and found that the frequencies of the genotypes were significantly different between patients with and without peri-implantitis. However, logistic regression showed that only the presence of the TNF-α genotype (−308) ( *p* = 0.003) can increase the risk of peri-implantitis, while the CD-14 polymorphism (−159) ( *p* = 0.002) is associated with a decreased risk. 7 This is explained by the functions of these immunoregulatory molecules, since in peri-implantitis, proinflammatory cytokines (TNF-α, IL-6) increase local secretion and metalloproteinase activity, resulting in stimuli for bone resorption mechanisms, and CD-14 plays an important role in immune defense against bacterial infection. 7 23

Divergent analyses were obtained by He et al, who in a Chinese population ( *n* = 144) verified the absence of associations between the TNF-α (−308) polymorphism ( *p* = 0.0745) and the risk of peri-implantitis, the greatest differences between the studies being the size of the samples and the criterion for excluding smoking patients in the latter article, 9 and by Gurol et al, who in a Turkish population ( *n* = 95) (16 with implant failure, 22 with chronic periodontitis, 23 with healthy implants for a minimum of 6 months, and 34 healthy controls) also showed no significant association of TNF-α (−308) ( *p* > 0.999) in the susceptibility to implant failure. 12 As for the IL-6 (−174) analyses, both the study by Petkovic-Curcin et al 7 and Melo et al 6 showed no correlation ( *p* = 0.0993 and *p* > 0.05, respectively).

Another IL studied and associated with the inflammatory immune response process is IL-4, 10 related to the secretion of immunoglobulins G and E, the lack of which can lead to an accumulation of macrophages, increased expression of CD-14, and high production of proinflammatory mediators, leading to bone resorption. 18 Pigossi et al found for the first time in a Brazilian population that the +33 C allele of the IL-4 gene polymorphism (+33) was associated with susceptibility to dental implant loss ( *p* = 0.0236), but that the IL-4 polymorphism (−590) was not associated with reduced survival, only with an increased risk of chronic periodontitis. Other single nucleotide polymorphism (SNP) variations can be investigated as haplotypes, and new results can be found regarding dental implant loss. 10

The role of IL-10 (−1082) and RANKL (−438) polymorphisms was investigated in a Brazilian population ( *n* = 90), as they could be involved in the success or failure of implants, directly affecting the balance between anti- and proinflammatory proteins. 8 No statistically significant difference was detected between implant failure and genotypes, concluding that there was no association between the two variables, 8 corroborating Petkovic-Curcin et al in their analysis of IL-10 (−1082) ( *p* = 0.309) 7 and Gurol et al 12 who, in addition to this specific polymorphism, studied the influence of IL-10 (−819) on implant failure, and both showed no significant association with reduced dental implant survival ( *p* > 0.999). Pigossi et al also revealed no relationship between dental implant loss and IL-10, with its three SNPs, IL-10 (−1082), IL-10 (−819), and IL-10 (−592), in a total of 277 patients, including 92 subjects with at least one implant loss. 1

Few comprehensive studies, including the types of altered SNPs in the gene related to reduced dental implant survival, were found. This literature review verifies results that are still controversial, which may be caused by the limited sample size, the definition of the term “dental implant failure” not yet fully elucidated in all the studies, and inhomogeneous inclusion and exclusion criteria, including ethnicity, age, and smoking. In many studies, there is no discrimination between the various types of allelic combinations that can be found, only indications of the presence or absence of genetic influence.

## Conclusion

In summary, the IL-1A (−889), IL-1B (+3954), and IL-4 (+33) polymorphisms had a stronger association with dental implant loss. Based on the complexity of the subject, more research is needed.
